# Non-cell autonomous and non-catalytic activities of ATX in the developing brain

**DOI:** 10.3389/fnins.2015.00053

**Published:** 2015-03-04

**Authors:** Raanan Greenman, Anna Gorelik, Tamar Sapir, Jan Baumgart, Vanessa Zamor, Michal Segal-Salto, Smadar Levin-Zaidman, Vassilis Aidinis, Junken Aoki, Robert Nitsch, Johannes Vogt, Orly Reiner

**Affiliations:** ^1^Department of Molecular Genetics, Weizmann Institute of ScienceRehovot, Israel; ^2^University Medical Center, Institute for Microscopic Anatomy and Neurobiology, Johannes Gutenberg-University MainzMainz, Germany; ^3^Central Laboratory Animal Facility, University Medical Center, Johannes Gutenberg-University MainzMainz, Germany; ^4^Department of Chemical Research Support, Weizmann Institute of ScienceRehovot, Israel; ^5^Division of Immunology, Biomedical Sciences Research Center ‘Alexander Fleming’Athens, Greece; ^6^Graduate School of Pharmaceutical Sciences, Tohoku UniversityMiyagi, Japan

**Keywords:** cortical development, radial glia, autotaxin, LPA, neuronal stem cell, *in utero* electroporation

## Abstract

The intricate formation of the cerebral cortex requires a well-coordinated series of events, which are regulated at the level of cell-autonomous and non-cell autonomous mechanisms. Whereas cell-autonomous mechanisms that regulate cortical development are well-studied, the non-cell autonomous mechanisms remain poorly understood. A non-biased screen allowed us to identify Autotaxin (ATX) as a non-cell autonomous regulator of neural stem cells. ATX (also known as ENPP2) is best known to catalyze lysophosphatidic acid (LPA) production. Our results demonstrate that ATX affects the localization and adhesion of neuronal progenitors in a cell autonomous and non-cell autonomous manner, and strikingly, this activity is independent from its catalytic activity in producing LPA.

## Introduction

How excitatory neurons reach their proper position in the developing brain has been the focus of intense research, since perturbations in this process have been shown to result in a wide spectrum of brain diseases, ranging from severe brain malformations, to diseases such as cognitive impairment and autism. Most of the molecular mechanisms known to control radial neuronal migration are cell autonomous and include for example proteins, which are involved in regulation of the cytoskeleton and cytoskeleton-associated motor proteins (reviews Ayala et al., 2007; Rakic et al., 2007; Jaglin and Chelly, 2009; Valiente and Marin, 2010; Reiner, 2013). Key examples of such proteins are LIS1 and DCX, where mutations of the corresponding genes in humans result in a brain malformation known as lissencephaly (Reiner et al., 1993; Des Portes et al., 1998; Gleeson et al., 1998, reviews Jaglin and Chelly, 2009; Valiente and Marin, 2010; Reiner, 2013; Reiner and Sapir, 2013). LIS1 is involved in regulation of microtubules and the microtubule associated molecular motor, cytoplasmic dynein, as well as regulation of the actin cytoskeleton through the activity of small GTPases (Faulkner et al., 2000; Niethammer et al., 2000; Sasaki et al., 2000; Smith et al., 2000; Kholmanskikh et al., 2003; Yamada et al., 2013) (review Reiner and Sapir, 2013). DCX is a microtubule and actin-associated protein, which interacts with cytoplasmic dynein and a member of the kinesin superfamily of proteins (Gleeson et al., 1999; Caspi et al., 2000; Kim et al., 2003; Tsukada et al., 2003, 2006; Gdalyahu et al., 2004; Schaar et al., 2004; Tanaka et al., 2004b; Bielas et al., 2007; Bechstedt and Brouhard, 2012; Liu et al., 2012). Despite these so-called cell autonomous functions, experimental evidence suggests that LIS1 (Hippenmeyer et al., 2010) and DCX (Bai et al., 2003) may also affect neighboring cells in a non-cell autonomous fashion.

To better understand the non-cell autonomous aspects of radial neuronal migration, we developed an *in vivo* assay in which migration defective cells, following treatment with either *Dcx* or *Dclk* shRNA, were isolated and subjected to microarray analysis. We identified mRNA encoding for secreted and transmembrane proteins, which were differentially expressed in the area where the impaired neurons clustered in the brain. While both shRNA treatments exhibited non-cell autonomous inhibition of neuronal migration, the morphology of the stalled cells differed between treatments. Comparison of the gene expression profile in both treatments revealed several differentially expressed genes, among which we detected autotaxin (ATX, also known as ENPP2, PD-Iα or lysoPLD).

Autotaxin is a secreted enzyme of 99 kDa, thus may fit to act in a non-cell autonomous way. It was originally identified as an autocrine factor, which stimulates tumor cell motility (Stracke et al., 1992). ATX becomes active and is secreted to the extracellular space following glycosylation and proteolytic cleavage of its N-terminal signal peptide (Jansen et al., 2005, 2007). ATX is a member of the ENPPs (ectonucleotide pyrophosphatase/phosphodiesterases) family. Each of the ENPPs contains a conserved catalytic domain, which hydrolyzes phosphodiester bonds of different nucleotides and phospholipids (Stefan et al., 2005). ATX is unique, as it is the sole member of the ENPPs that utilizes this catalytic domain for lysophospholipase D (lysoPLD) activity. ATX catalyzes lysophosphatidic acid (LPA) production from lysophosphatidylcholine (LPC) (Tokumura et al., 2002; Umezu-Goto et al., 2002). ATX is considered as the major producer of LPA, and deletion of one allele reduces LPA concentration in the plasma by half (Tanaka et al., 2006; Van Meeteren et al., 2006). Thus, it is thought that ATX acts predominantly through LPA production. LPA is a potent molecule, which acts through binding to its cognate receptors (LPAR1-5) thus instigating several downstream signaling pathways. Nevertheless, single LPAR knockout mice develop normally. LPA influences multiple events during cortical development including polarity establishment in hippocampal neurons (Yamane et al., 2010). In addition, LPA regulates proliferation, survival and differentiation in sundry cell populations. Heuristically, physiological concentrations of LPA (0.1 ~ 1 μM) promote proliferation of several neuronal progenitors and stem cells and enhance cortical growth (Kingsbury et al., 2003; Fukushima, 2004; Svetlov et al., 2004; Cui and Qiao, 2006; Estivill-Torrus et al., 2008; Hurst et al., 2008), while higher concentrations of LPA evoke necrosis and apoptosis (Holtsberg et al., 1998; Steiner et al., 2000). LPA has been shown to be a survival factor of neuroblasts (Kingsbury et al., 2003) and post-mitotic neurons (Fujiwara et al., 2003; Zheng et al., 2005; Estivill-Torrus et al., 2008). LPA has been shown to stimulate both neuronal differentiation, possibly through LPAR1 (Cui and Qiao, 2006; Fukushima et al., 2007; Spohr et al., 2008), and glial differentiation (Cui and Qiao, 2007), yet other studies suggest that LPA inhibits neuronal differentiation (Dottori et al., 2008). In mice, ATX knockout is lethal and embryos die around E9–E10 (Tanaka et al., 2006; Van Meeteren et al., 2006; Fotopoulou et al., 2010). These mice display vascular defects in embryo and yolk sac, allantois malformation, neural tube defects, asymmetric headfolds, increased cell death, decreased proliferation and neurite outgrowth deficits. Neurite outgrowth was rescued by addition of LPA (Fotopoulou et al., 2010). Heterozygous-knockout mice, exhibiting half of the lysoPLD activity and LPA levels, showed attenuated nerve injury-induced neuropathic pain (Inoue et al., 2008). High ATX expression, on the other hand, is associated to and found in many pathophysiological conditions, including several cancer types (Okudaira et al., 2010), neuropathic pain (Inoue et al., 2004, 2008; Ueda, 2008), Alzheimer-type dementia (Umemura et al., 2006), multiple sclerosis (Hammack et al., 2004) and following brain lesion (Savaskan et al., 2007). During embryonic development, ATX expression is first detected at the floor plate of the neural tube, and later in the choroid plexus, cerebrospinal fluid and the ventricular area of the embryonic brain (Abramova et al., 2005; Ohuchi et al., 2007; Savaskan et al., 2007; Zappaterra et al., 2007). Following birth, ATX is detected in leptomeningeal cells, oligodendrocytes and astrocytes, but not in neurons. ATX induces neurite retraction of differentiated PC12 via LPA production (Sato et al., 2005). In oligodendrocytes ATX is upregulated during maturation and is temporally correlated with the process of myelination. ATX facilitates morphological changes of oligodendrocytes, decreases their adhesion to the ECM and promotes complex process network (Fox et al., 2004; Dennis et al., 2008, 2011). Little is known about the role of ATX during cortical development. Our studies show cell-autonomous and non-cell autonomous roles of ATX in regulation of cell position and adhesion in progenitors of the developing cortex. Markedly, these activities did not require ATX catalytic activity.

## Materials and methods

### Animals

ICR were purchased from Harlan laboratories. Mice in which the first two exons of *ATX* gene are flanked by two loxP sites were obtained from Vassilis Aidinis (Fotopoulou et al., 2010) and were bred with mice which express the recombinase Cre under the control of the *EMX1* promoter (Jackson). Genotyping for the *Atx*^flox^ and *Atx*^−^ alleles was described previously (Fotopoulou et al., 2010). Briefly, four primers were used: A1, B1, C1, and B2. A1: 5′-CGCATTTGACAGGAATTCTT; B1: 5′-ATTTGTCACGTCCTGCACGA; C1: 5′-ATCAAAATACTGGGGCTGCC; B2: 5′-TACACAACACAGCCGTCTCA. Primer combination A1 and C1 was used to detect wild type (WT) alleles. Primer combination A1 and B1 was used to detect the floxed (neo) allele. Primer combination A1 and B2 was used to detect the deleted allele. The primers used for detecting the *EMX1-Cre* transgene were 5′-AACATGCTTCATCGTCGG and 5′-TTCGGATCATCAGCTACCACC. Embryonic day 0 (E0) was defined as the day of confirmation of the vaginal plug. Mice were raised in the Weizmann Institute of Science transgenic facility. All animal procedures were approved by IACUC.

### Immunohistochemistry

Antibodies used were as follows: mouse anti-5-iodo-2′-deoxyuridine(IdU)/5-bromo-2′-deoxyuridine(BrdU) (1:50; BD Biosciences), rat anti-BrdU (1:100; Becton Dickinson), rabbit anti-phosphorylated histone H3 (pH3) (1:100; Upstate Biotechnology), goat anti-GFP (1:400; Abcam), chicken anti-GFP (1:500; Abcam), rat anti-ATX [1:40; kindly provided by J. Aoki (Tanaka et al., 2004a)], chicken anti-Tbr2 (1:400; Millipore), chicken anti-Tbr1 (1:400; Millipore), mouse anti-Tuj1 (1:300; Covance), goat anti-Par-3 (1:50; Santa Cruz Biotechnology), rabbit anti-β-Catenin(1:300; Sigma), rat anti-ZO-1(1:70; Developmental Studies Hybridoma Bank), mouse anti-Numb (1:300; Developmental Studies Hybridoma Bank), goat anti-Par-6 (1:100; Santa Cruz Biotechnology), rabbit anti-Pax6 (1:300; Covance), pFAK 925 (1:100; Cell Signaling).

Floating sections or cryosections were permeabilized using 0.1% Triton X-100 and blocked in blocking solution (PBS, 0.1% Triton X-100, 10% HS; or PBS, 0.1% Triton X-100, 2% HS for ATX staining) for 60 min. Antibodies were incubated in blocking solution over night at 4°C. After washing, appropriate secondary antibodies (Jackson ImmunoResearch) were diluted in blocking solution, and incubated for 2–3 h at room temperature. Slices were mounted onto glass slides using Aqua Polymount (Polysciences).

Cover slips containing fixed cells were permeabilized using 0.1% Triton X-100 and blocked three times in PBS supplemented with 0.1% BSA (Sigma). Coverslips were incubated with antibodies, stained with DAPI and mounted onto glass slides using Aqua Polymount (Polysciences). To visualize ATX, sections were first incubated with 10 mM citrate buffer for 30 min in 80°C, then cooled at RT for 30 min. After washing, sections were immunostained as described above.

### Analysis of neuronal morphology

The z-stack images from the slices of the *in utero* electroporated (E14.5–E18.5) brains with either *Dcx* or *Dclk* shRNA were acquired with confocal microscope (LSM480, Zeiss, x40). Each fluorescent cell in the resulted images was classified as either bipolar, cells with 3–4 processes or multipolar. Slices from four different brains for each condition were used for the analysis. In total 188 and 212 cells from *Dcx* and *Dclk* shRNA condition respectively were analyzed.

### Sample preparation and microarray analysis

*In utero* electroporation was performed on E14.5 mouse brains with *Dcx* or *Dclk* shRNA together with GFP in 3:1 ratio. On E17.5 the mice were sacrificed and the embryos collected in L-15 (Biological Industries) supplemented with gentamycin, glucose (0.6%) and saturated with oxygen in RNase-free environment. The fluorescent area of the cortex was cut out with a razor under the fluorescent binocular and homogenized in TRI Reagent (Sigma, Israel). After addition of 0.2 ml of chloroform per 1 ml of TRI Reagent used, the samples were mixed and centrifuged at 13000 rpm for 15 min at 4°C. The upper aqueous phase was precipitated with 0.5 ml Isopropanol. The precipitated RNA was washed with 70% Ethanol, dissolved in water, and cleaned with RNeasy Mini Kit (Qiagen). The Mouse Gene 1.0 ST Array was used for Affymetrix analysis. The experiments were repeated twice, and each repeat was composed of a RNA pool derived from 4 to 6 electroporated brains. The correlation between the repeats was very high (*R*^2^ = 0.9955 and 0.9925 for *Dcx* and *Dclk* shRNA conditions, respectively). Only genes that showed at least 1.9 fold-difference of expression were selected for further analysis.

### Plasmids and RNAi constructs

ATX shRNA1 and shRNA2 are pLKO.1 lentiviral shRNA constructs purchased from Open BioSystems (TRCN0000080829 and TRCN0000080830, respectively). The experiments shown in the figures are corresponding to shRNA2, but most experiments were conducted using both shRNA sequences in parallel and no differences were noted. Control shRNA was previously described (Sapir et al., 2012).

The full-length human ATX (hATX) was provided from Prof. Junken Aoki (Hashimoto et al., 2012). The full-length rat ATX (rATX) was provided from Prof. Mathieu Bollen (Jansen et al., 2005), and subcloned into pCAGGS vector using the NheI and NotI restriction sites. Site-directed mutagenesis of the catalytic domain (T210A) in hATX and rATX were performed using the primers 5′-TCCCTACATGAGGCCGGTGTACCCAACTAAAgCCTTTCC and 5′-GCCTCTGGTGAAGAGCTCAG for hATX, and 5′-CTGTGTACCCCACAAAAgCCTTCCCTAATC and 5′-GATTAGGGAAGGcTTTTGTGGGGTACACAG for rATX. The PCR product of the mutant hATX was subcloned into full-length hATX using the EcoRI and EcoNI restriction sites. The mutant hATX contained additional mutations T241S, V279S, T294S, H298N; all of which are conserved in mouse and rat. The mutant rATX contained an additional mutation at the linker region (L581F). Plasmids were co-electroporated with a fluorescent protein. Co-electroporation of pCAGGS-GFP, pCAGGS-mCherry was performed for the *in utero* and *ex utero* electroporation experiments. Co-electroporation of pCAGGS-GFP, EF-LPL-lynGFP, Tα-LPL-GAP43-Strawberry, Tα-Cre (provided from Prof. Akira Sakakibara) and PGK-Cre was performed for the lattice culture and flow cytometry experiments. FUCCI cell cycle reporters (Sakaue-Sawano et al., 2008) were subcloned into pCAGGS.

### *In utero* electroporation

Plasmids were transfected by *in utero* electroporation using previously described methods (Sapir et al., 2008). Briefly, E14 or E13 pregnant female ICR mice were anesthetized by intraperitoneal injection of 10% ketamine/20 mg/ml xylazine (1/10 mixture, 0.01 μl/g of body weight, i.p.), alternatively isoflurane anesthesia was utilized. The uterine horns were exposed, and plasmids (0.5–1 μl) mixed with Fast Green (2 μg/μl; Sigma) were microinjected by mouth pipette through the uterus into the lateral ventricles of embryos by pulled glass capillaries (Sutter Instruments). Electroporation was accomplished by delivering five electrical pulses (50 ms duration) at intervals of 950 ms with a square-pulse electroporator (Nepa Gene), using a platinum-plated tweezer electrodes (Protech International).

For knockdown or overexpression, a GFP expression vector with either shRNA, ATX, or mutant ATX expression vector (3:1 ratio) were used. For rescue experiments, equal amounts of ATX shRNA2 and either hATX or mutant hATX were used. Cell cycle analysis was performed by *in utero* electroporation with FUCCI reporters.

For the analysis of cell location, morphology and type, embryos were intracardially perfused using 4% paraformaldehyde–phosphate buffered saline (PFA-PBS). Brains were post-fixed overnight and sectioned (60 μm; vibrotome, Leica).

### *Ex utero* electroporation

E14 embryos were removed from pregnant dams. DNA mixtures (equal concentrations as used for the *in utero* electroporation) were injected to the ventricles and electroporation was conducted by delivering five electrical pulses (50 ms duration) at intervals of 950 ms with a square-pulse electroporator (Nepa Gene), using 5-mm-diameter platinum-plated tweezer electrodes (Protech International). Brains were removed in cold L-15 (Biological Industries) supplemented with gentamycin, glucose (0.6%) and saturated with oxygen. Freshly isolated whole brains were cut into 250 μm coronal slices and then transferred onto inserts (MilliCell-CM; 0.4 μm; Millipore) floating on 1 ml of either serum-free medium (Neurobasal medium supplemented with B27, N2, GlutaMax, glucose, and gentamicin) or condition-medium (described below in cell-culture and condition media). Brain slices were cultured at 37°C and 5% CO_2_ for 2 days. Half of the media was replaced with fresh media after 24 h. Slices were fixated with 4% PFA-PBS overnight, incubated at 4°C in PBS/30% sucrose, frozen on dry ice with OCT compound and cryosectioned (10 μm; Leica CM3050S).

### Cell-culture and conditioned media

Conditioned media for *ex utero* experiments were prepared using HEK293 cell line overexpressing either GFP and ATX or mutant ATX. Cells were grown at 37°C and 5% CO_2_ in MEM (Dulbecco's modified Eagle's medium supplemented with 5% fetal calf serum, 5% horse serum, B27, Glucose, GlutaMax and Gentamicin). Media was collected 2 days following calcium-phosphate transfection (Graham and Van Der Eb, 1973) of 2 μg DNA. Collected media was diluted 1:3 in fresh MEM and kept in 4°C for 2–5 days.

#### Microscopy

Images were taken either with wide-field microscopy with the DeltaVision system package (Applied Precision, Issaquah, WA, USA), Pannoramic MIDI scanner (3DHisthech) or by confocal microscopy (LSM510, Zeiss, LSM 780).

#### Analysis

Cell counts were analyzed using the spots module of Imaris software (Bitplane, Zurich, Switzerland). Intensity and circularity were measured using the ImageJ software (NIH).

Statistical analysis was conducted using Prism 5 for Macintosh (GraphPad Software, Inc.).

## Results

### Screening for non-cell autonomous factors involved in radial migration

**Figure 1:**
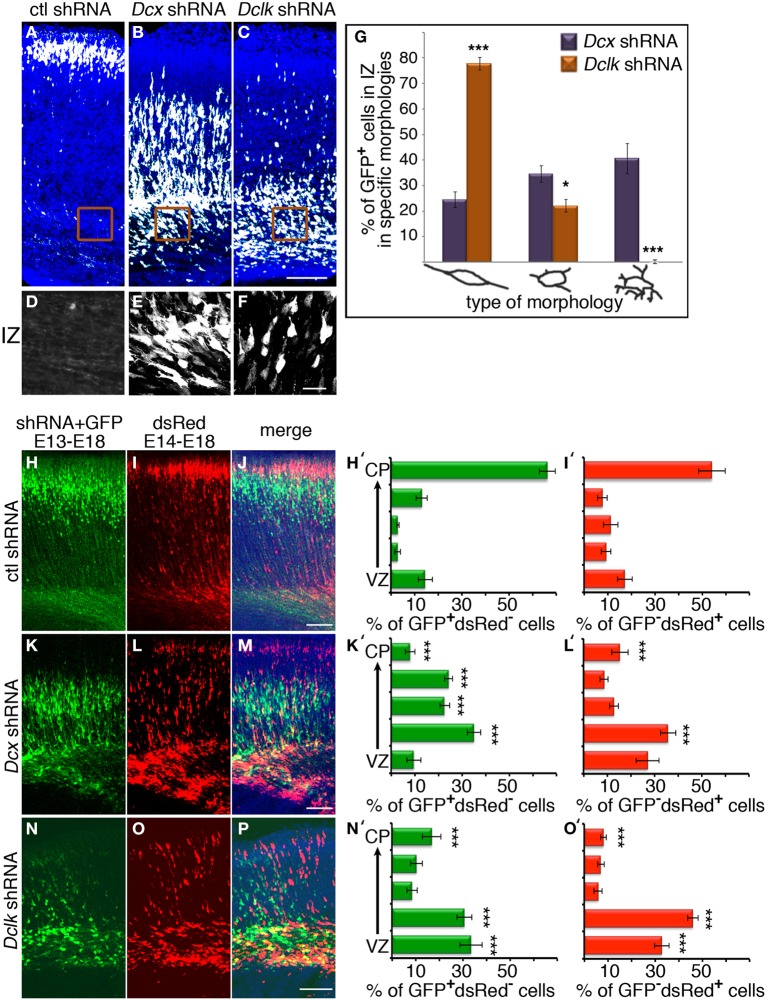
**Cell autonomous and non-cell autonomous effects of *Dcx* and *Dclk* on neuronal migration. (A–C)** *Dcx* and *Dclk* impair radial neuronal migration. Brains electroporated *in utero* on E14 with control **(A)**, *Dcx* **(B)** or *Dclk* **(C)** shRNA constructs together with GFP, were analyzed on E18. Scale bar, 100 μm. **(D–F)** The morphology of cells arrested in the IZ at **(E)** with reduced DCX **(E)** or DCLK **(F)**. No cells in the IZ were observed in control shRNA. Scale bar, 20 μm. **(G)** Quantification of cells arrested in the IZ exhibiting bipolar, multipolar and highly branched multipolar morphologies from sections from four different brains of each treatment (*Dcx* and *Dclk* shRNA). *Student t-test* ^*^p < 0.05; ^***^p < 0.001. **(H–P)** Non-cell autonomous effect on migration of *Dcx* and *Dclk* shRNA. Brains were *in utero* electroporated with control shRNA **(H–J)**, *Dcx* shRNA **(K–M)** or *Dclk* shRNA **(N–P)** together with GFP on E13, followed by electroporation with dsRed on E14. The analysis was performed on E18. GFP-positive cells **(H,K,N)**, dsRed-positive cells **(I,L,O)** and merged images **(J,M,P)** are shown. Only single positive cells were counted and used for quantifications and statistical analysis. In each section the total number of cells were considered 100% and the relative % of green and red cells were calculated in relation to the same population. In the histograms the % of GFP positive dsRed negative or % of GFP negative dsRed positive cells positioned in the different bins are indicated. The statistical analysis is based on number of cell bodies that were counted in five arbitrary bins spanning the width of the cortex. The analysis was done using the Imaris© software **(H′,I′,K′,L′,N′,O′)**. Scale bar, 100 μm.

**Figure 2:**
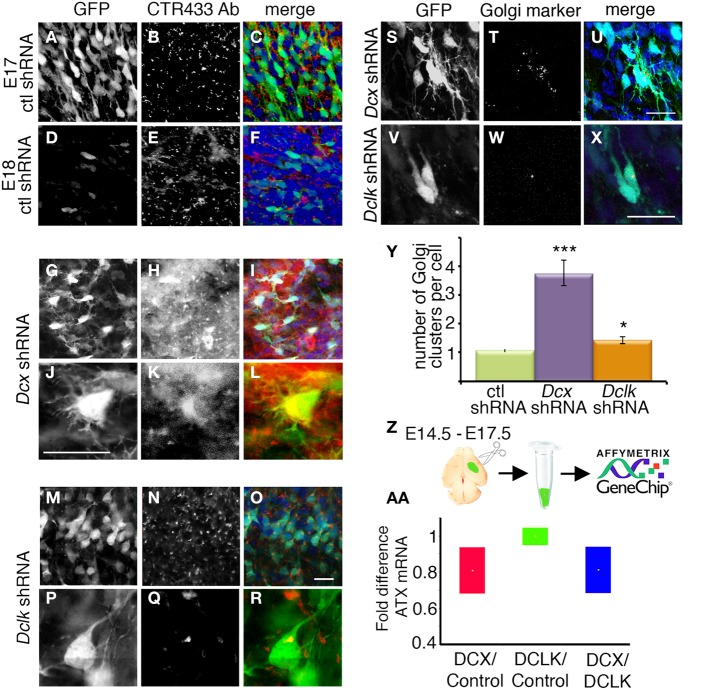
**(A–X)** The organization of the Golgi apparatus in *Dcx* and *Dclk* shRNA transfected neurons stalled in the IZ. **(A–R)** Golgi organization is shown by Golgi specific immunostaining. Control **(A–F)**, *Dcx* **(G–L)** or *Dclk* **(M–R)** shRNA were electroporated brains (E14–E18) were immunostained with CTR433 Golgi antibodies. Since there are practically no cells in the area of interest (IZ) in the control experiment, an additional control was used, in which the control shRNA was electroporated from E14 to E17, when some control cells still reside in the IZ **(A–C)**. GFP **(A,D,G,J,M,P)** serves as a marker for electroporated cells. **(S–X)** Verification of Golgi organization shown by coelectroporation with a plasmid expressing a Golgi marker. *Dcx* **(S–U)** or *Dclk* **(V–X)** shRNA were coelectroporated with a Golgi marker plasmid, 82 amino acids of β1,4-Galactosyltransferase fused to mCherry. GFP **(S,V)** serves as a marker for electroporated cells. **(Y)** Quantifications of Golgi clusters per cell was performed on control (E17), *Dcx* or *Dclk* shRNA treated cells (an average of 20 cells was used for quantifications and statistical analysis) ^*^p < 0.05, ^***^p < 0.001. **(Z)** Experimental design of the Affymetrix GeneChip experiment. Embryos were electroporated *in utero* on E14; on E17 the areas where stalled electroporated cells were dissected. RNA or protein was extracted from 5 embryos per experiment. **(AA)** Real-Time PCR validation of Affymetrix Gene Chip experiment. The *Atx* mRNA levels in the *Dcx* shRNA treated brain were reduced by 40% in comparison to *Dclk* shRNA or in comparison to control shRNA in concordance with Affymetrix results. Scale bars: panels 20 μm.

Knockdown of *Dcx* and *Dclk* was reported to impair radial neuronal migration (Bai et al., 2003; Koizumi et al., 2006; Ramos et al., 2006). In line, we could show that *Dcx* or *Dclk* knockdown impaired cell migration (Figures 1A–F). Although reduction of DCX induced cells to arrest with multipolar morphology (Bai et al., 2003) (Figures 1B,E,G), cells treated with *Dclk* shRNA exhibited bipolar morphology (Figures 1C,F,G). To investigate cell autonomous and non-cell autonomous effects of a particular intervention we modified a previously described approach (Bai et al., 2003). The experimental design included labeling and monitoring two distinct populations in the developing embryonic brain by consecutive electroporation. The first population was treated with shRNA (at day E13) and labeled with GFP. We have confirmed that no plasmid that is injected in the early timepoint lingered in the ventricle (data not shown). The position of the first population reflected cell autonomous effects. The second cell population was electroporated with a red fluorescent protein expression construct only a day later (E14) and reflected non-cell autonomous effects emanating from the first (green) population. *Dcx* shRNA treatment inhibited neuronal migration in a cell autonomous way (Bai et al., 2003) (control shRNA treated green cells in Figures 1H,J in comparison with *Dcx* shRNA treated Figures 1K,M quantified in Figures 1H′,K′ respectively) as well as in a non-cell autonomous fashion (Bai et al., 2003) (dsRed labeled cells in Figures 1I,J in comparison with Figures 1L,M quantified in Figures 1I′,L′ respectively). Likewise, *Dclk* shRNA treatment affected neuronal migration in a cell autonomous and non-cell autonomous fashion (Figures 1N–P). Therefore, we conclude that both *Dcx* and *Dclk* shRNA treatments affect the position of the transfected cells themselves in a cell autonomous and in addition, the transfected cells affect neighboring cells, born a day later, in a non-cell autonomous way. The distribution of the Golgi within the cell was used as a marker for its polarization. Control cells showed compact Golgi either at E17 (where more cells can be detected at the SVZ/IZ border) or E18 (Figures 2A–F respectively). However, cells treated with *Dcx* shRNA but also their neighboring cells displayed dispersed Golgi (Figures 2G–I, higher magnification Figures 2J–L, quantified in Figure 2Y), yet the Golgi appeared compact in cells treated with *Dclk* shRNA and their neighbors (Figures 2M–O, higher magnification Figures 2P–R, quantified in Figure 2Y). In addition, to better visualize cell autonomous effects on the Golgi, brains were co-electroporated with the corresponding shRNA, GFP and a Golgi marker. In case of *Dcx* shRNA the Golgi was dispersed (Figures 2S–U) and in case of *Dclk* shRNA the Golgi was compact (Figures 2V–X). Therefore, it was possible to visualize that *Dcx* shRNA treated cells exhibit abnormal polarity, as revealed by dispersed Golgi staining, and also the untreated neighboring cells exhibited abnormal polarity. Since both shRNA treatments affected cell migration in a non-cell autonomous fashion, while the stalled cells exhibited different states of cell polarization, we set out to identify differentially expressed genes in the cells residing in the stalling area. Areas enriched with stalled fluorescent cells were dissected out at day E17 from brains, which had been electroporated *in utero* at day E14. The extracted mRNA was converted to cDNA and subjected to Affymetrix chip analysis (scheme in Figure 2Z). This approach identified a few distinct genes that differed in their expression levels between the *Dcx* and *Dclk* shRNA-treated cells (Table 1).

**Table 1:** **The analyzed results of the Affymetrix experiment**.

**Gene name**	**Fold difference**	
**(A) UNKNOWN GENES: HIGHER TRANSCRIPTION LEVEL IN *Dclk* shRNA TREATED EMBRYOS**		
ENSMUSG00000074558	4.42	Predicted gene encoding protein with 5 TM domains, a member of ENSFM00360000113264 gene family
ENSMUSG00000074562	9.51	Predicted gene encoding protein with 5 TM domains, a member of ENSFM00360000113264 gene family
ENSMUSG00000074566	9.15	Predicted gene encoding protein with 5 TM domains, a member of ENSFM00360000113264 gene family
ENSMUSG00000075014	4.31	Predicted gene encoding protein with 5 TM domains, a member of ENSFM00360000113264 gene family
ENSMUSG00000058736	3.29	Putative gene encoding secreted peptide
**(B) GENES WITH HIGHER TRANSCRIPTION LEVEL IN *Dclk* shRNA TREATED EMBRYOS**		
*Ctsc*	2.02	Cathepsin C
*Enpp2*	1.95	Ectonucleotide pyrophosphatase/phosphodiesterase 2
*Ifitm3*	2.70	Interferon induced transmembrane protein 3
*Serping1*	2.10	Serine (or cysteine) peptidase inhibitor, clade G, member 1
*Ttr*	7.02	Transthyretin
**(C) GENES WITH HIGHER TRANSCRIPTION LEVEL IN *Dcx* shRNA TREATED EMBRYOS**		
*Gcg*	2.04	Glucagon
*Penk*	2.05	Preproenkephalin
*Tcfap2d*	2.30	Transcription factor AP-2, delta
*Zfp125*	2.47	ZT2 gene encoding zinc finger protein 125

*The genes with different transcription level between Dcx and Dclk shRNA electroporated brain regions are divided into 3 categories: unknown genes (A), known genes with higher transcription level in Dclk shRNA condition (B), and known genes with higher transcription level in Dcx shRNA condition (C). Averages for fold differences of 2 biological repeats are shown*.

Results identified 14 novel genes, most of which encode secreted or extracellular proteins, suggesting an involvement of non-cell autonomous mechanisms. Of particular interest, *Enpp2*, Ectonucleotide Pyrophosphatase/Phosphodiesterase 2, PD-Iα or lysoPLD, also known as *Autotaxin (Atx)* had a twofold expression in the bipolar *Dclk* shRNA treated neurons. This result was reconfirmed using real-time qPCR (Figure 2AA) and Western blot analysis (data not shown). We therefore focused on the cell autonomous and non-cell autonomous roles of *Atx* in the developing brain.

### ATX is expressed in the developing brain

**Figure 3:**
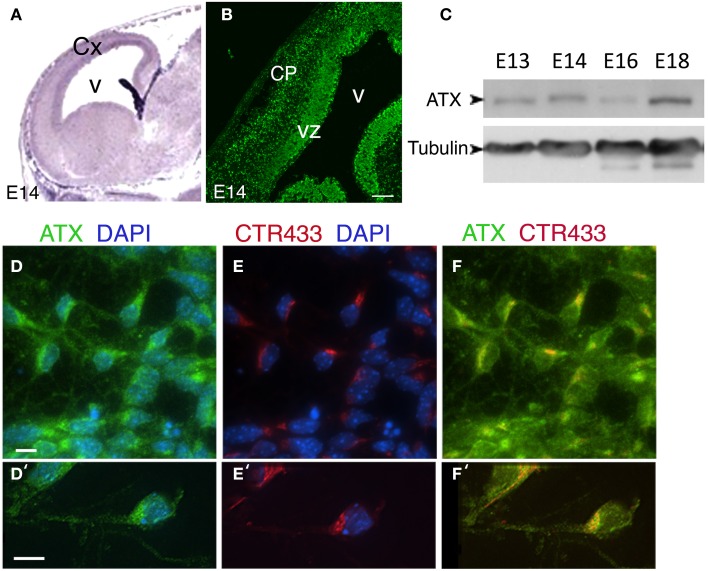
**Expression and localization of ATX in the developing mouse cortex. (A)** *In situ* hybridization of *Atx* in the developing mouse cortex at E14 (from http://www.genepaint.org/). **(B)** Coronal cryosections of the mouse embryonic cortex at E14 immunostained for ATX. **(C)** ATX levels in cortical lysates at different developmental stages. α-tubulin was used as a loading control. **(D–F′)** Cultured cortical neurons isolated from E14 mouse cortices were grown 3 DIV, immunostained for ATX (green) **(D–F, D′–F′)** and the Golgi marker CTR433 (red) **(E,E′)**, and counterstained with DAPI. Scale bars: **(A,B)** 100 μm, **(D′–E)** 10 μm. CP, cortical plate; Cx, cortex; V, ventricle; VZ, ventricular zone.

Previous studies indicate that *Atx* mRNA is expressed in the choroid plexus and the ventricular zone (VZ) during cortical embryonic development (Ohuchi et al., 2007; Savaskan et al., 2007) (Figure 3A, from http://www.genepaint.org/). Immunostaining of mouse E14 brain sections using a previously characterized monoclonal antibody (Tanaka et al., 2004a), revealed strong expression in the VZ, but also in the cortical plate (CP) (Figure 3B). ATX protein is expressed throughout cortical development, as demonstrated by Western blot analysis (Figure 3C). The subcellular localization of ATX was analyzed using E15 dissociated cortical neurons. ATX was expressed by all neurons. Notably, most of the protein was localized perinuclear in vesicular structures (Figures 3D–F,D′–F′), and colocalized with the Golgi apparatus, immunostained with CTR433 antibodies (Figures 3E,E′). Part of the protein was noticed in the growing neurites.

### ATX affects cell adhesion in the ventricular zone

**Figure 4:**
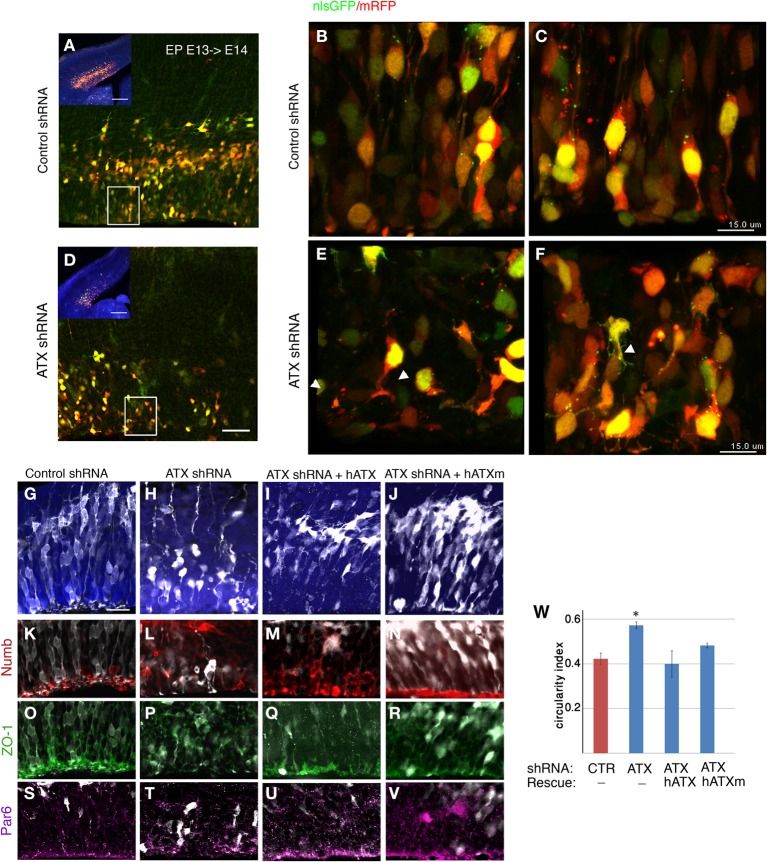
**ATX knockdown affects the radial progenitors adhesion and VZ polarity. (A–E)** coronal sections from E14 electroporated brains. E13 embryos co-electorporated with control shRNA, dsRed and NLS-GFP **(A–C)** or with *Atx* shRNA **(D–F)**. Lower magnification is shown in **(A,D)**. White rectangle in panels **(A,D)** indicate area magnified in **(B)** and **(E)** respectively. White arrowheads point at endfeet that are not tethered to the apical surface of the VZ following ATX shRNA introduction. **(C–H)** ATX knockdown impairs polarity at the VZ and affects organization and morphology of cells. **(G–V)** E15 brains that had been electroporated at E14 are depicted. Whereas in the control brains **(G)** most of the cells (white, GFP) exited the VZ, upon ATX knockdown **(H)** cells were generally reside within the VZ. ATX knockdown distrups the structure of the VZ. ATX knocked-down cells exhibited a long and crooked radial process, and are much rounded than control cells. These knockdown effects were rescued with the co-electroporation of the *Atx* shRNA and human ATX cDNA (hATX), which is resistant to the Atx shRNA **(I)**. Partial rescuing effect was seen with the co-electroporation of a mutated non-catalytic human ATX (hATXm, **J**). Immunolabeling of the apical polarity markers Numb **(K–N)**, ZO-1 **(O–R)** and Par6 **(S–V)**, demonstrated a loss of polarized localization of these protein in the ATX knockdown brains. The localization was restored upon introduction of hATX as well as non-catalytic ATX. **(W)** Changes in cellular roundness were measured and analyzed using the circularity index. Data are presented as mean±SEM; *n* = 3 brains for each condition. ^*^p < 0.05 (Kruskal-Wallis test followed by Dunn's Multiple Comparison Test). Scale bars: **(A,D)** (insert) 200 μm, **(A,D)** 50 μm, **(C,F)** 15 μm **(G)** 25 μm, **X**(upper panel) 50 μm, **X**(lower panel), 25 μm.

**Figure 5:**
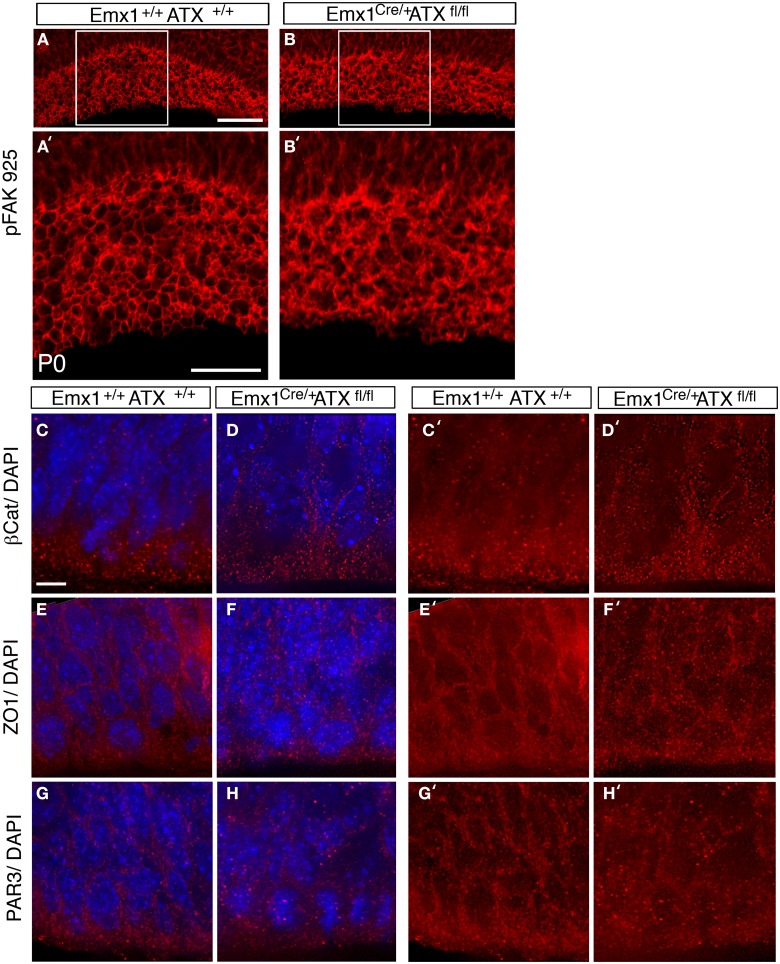
**ATX knockout have subtle effects on adherence junctions in the apical VZ. (A–B′)** Disruption in pFAK 925 immunostaining is observed in ATX knockout mouse. **(B,B′)** when compared to floxed allele carrier, non-deleted littermate at P0 **(A,A′)**. **(C–H′)** Staining with adherence junction markers, β Catenin **(C–D′)** ZO1 **(E–F′)** and with the apical polarity marker Par3 **(G–H′)** in brains of E14 wt **(C,C′,E, E′,G,G′)** and mutant littermates **(D,D′,F,F′,H, H′)**. All three antigens accumulated at the cell–cell contact sites in both wt and mutant brains sections. Size markers: **(A)** 50 μm, **(A′)** 25 μm **(C)** 3 μm.

**Figure 6:**
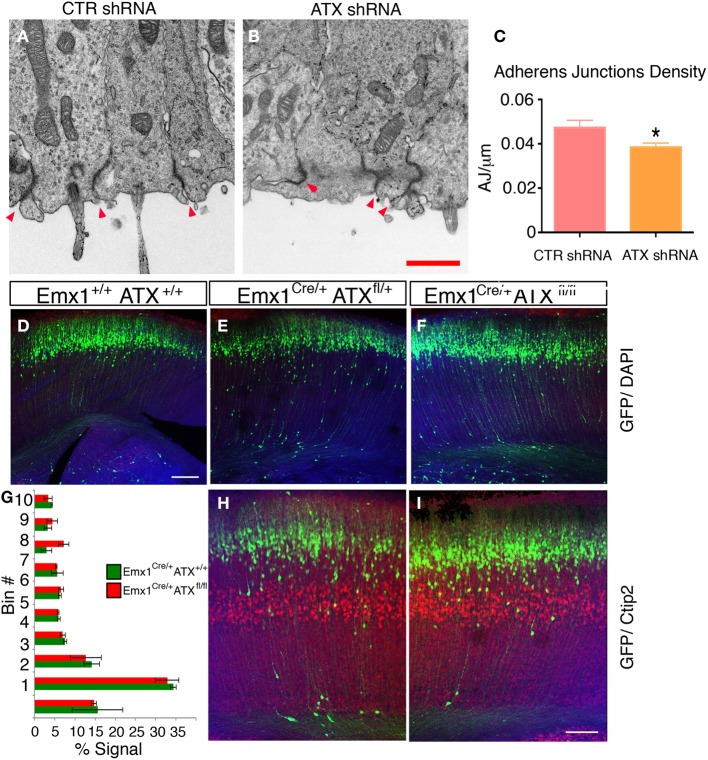
**(A–C)** ATX Knockdown has minor effect on adherence junctions density in the apical aspect of the ventricular zone. **(A,B)** Representative electron micrographs of ventricular zones of Control shRNA **(A)** or ATX shRNA **(B)** treated brains. The sections were obtained from E14 brains, electroporated a day earlier. The areas imaged were identified as electroporated by a GFP signal (not shown), prior to preparation for imaging. Adherence junctions are the dense areas decorating the border between adjacent cells, red arrowheads **(C)** Adherence Junctions density as measured from (7 to 15) electron micrographs recorded from two brains per treatment, ^*^p < 0.05. **(D–I)** ATX Knock out embryos do not display a neuronal migration phenotype. **(D–F)** littermates of indicated genotypes were electroporated *in utero* at E14 with GFP expressing plasmid and analyzed 4 days later. The location of the GFP+ cells did not differ in WT **(D)** heterozygous **(E)** or ATX knockout embryos **(F,G)** The distribution of GFP+ cells along 10 arbitrary bins spanning the width of the cortex in mutant (Red) and WT (green) embryos are presented. The shown results are averaged from 3 to 4 brains. **(H,I)** Deeper layers neurons (Ctip2+, layer V) are normally layered in WT **(G)** and Knockout **(I)** E18 embryos. Size markers **(F)** 100 μm, **(H)** 1 μm.

To examine the effect of ATX reduction in the developing brain, we *in utero* electroporated brains with either *Atx* shRNA or control shRNA at E13 and examined them at E14 (Figures 4A–F). Real-time PCR indicated that the shRNA reduced *Atx* mRNA levels to 30.8 ± 5.2% (*n* = 3 ±S.D.) in comparison with control. Cells with reduced ATX levels demonstrated distorted morphology, most of the cells were round, and in some cases the endfeet were not tethered to the apical aspect of the ventricular zone (Figures 4E,F). These results were recapitulated when brains were *in utero* electroporated at E14 and analyzed at E15 (Figures 4G–V). To gain additional information regarding the observed phenotype, the brain sections were immunostained with the apical markers Numb, ZO-1 and Par6. As expected, these proteins were apical in control brain sections, however, they displayed abnormal positioning in shRNA treated sections. Immunostaining of treated brain sections with β-catenin and Par3 antibodies did not reveal any changes in the localization of these proteins in the ATX knockdown brains (data not shown). Cellular morphology and the proper positioning of Numb, ZO-1 and Par6 were largely restored following the addition of human ATX expression construct, which is resistant to the shRNA (Figures 4M,Q,U). Surprisingly, introduction of the catalytically inactive human ATX expression construct was able to restore these observed phenotypes (Figures 4N,R,V). The measured circularity index statistically differed from control only with the sole addition of *Atx* shRNA (Figure 4W), while the addition of ATX or mutant ATX resulted in an elongated morphology and the recurrence to control circularity levels. To confirm the results obtained by knockdown experiments we used a genetic model. We therefore examined the cellular localization of several proteins in floxed *Atx* mice deleted with *Emx1*-drived Cre. Adherens junctions were immunostained using phosphorylated FAK antibodies (Figures 5A–B′). Images were acquired from thick sections show that adherence junctions form from the ventral side of the ventricular zone in control brains, while in ATX depleted brains adherens junctions were somewhat distorted, recapitulating our findings in the acute knock down experiments. Additional immunostainings with β-catenin (Figures 5C–D′), ZO1 (Figures 5E–F′) and Par3 (Figures 5G–H′) did not reveal very striking differences. To further explore the adhesion junctions following *in utero* electroporation, we conducted electron microscopy analysis on sections from treated brains (Figures 6A–C). The presence of adherens junctions is obvious in the control shRNA and *Atx* shRNA sections (Figures 6A,B, marked with red arrowheads). Quantitative analysis revealed a slight reduction in the density of the adhrens junctions in the *Atx* shRNA treated sections (Figure 6C). A possible effect on cellular adhesion may involve proteins such as N-cadherin and E-cadherin that are normally accumulated at the ventricular surface. N-cadherin is known to regulate neuronal migration as well as ventricular structures (Kawauchi et al., 2010; Jossin and Cooper, 2011). Therefore, we examined the possibility that Atx knockout neurons exhibit neuronal migration deficits (Figures 6D–I). *In utero* electroporation of a GFP expression plasmid at E14 and analysis at E18 of wildtype embryos (Emx1-Cre negative) (Figure 6D), heterozygote for the floxed allele (Emx1-Cre positive Atx1 fl/+), and homozygote for the floxed allele (Emx1-Cre positive Atx1 fl/fl), exhibited no obvious differences (quantified in Figure 6G). The position of CTIP2 positive cells, which label layer 5, did not differ between the heterozygotes and the mutant mice (Figures 6H–I). Overall, our results suggest that ATX affects cell adhesion in the ventricular zone and this activity is in part not dependent upon its enzymatic activity.

### ATX affects cell positioning in the ventricular zone in a cell autonomous and non-cell autonomous way

**Figure 7:**
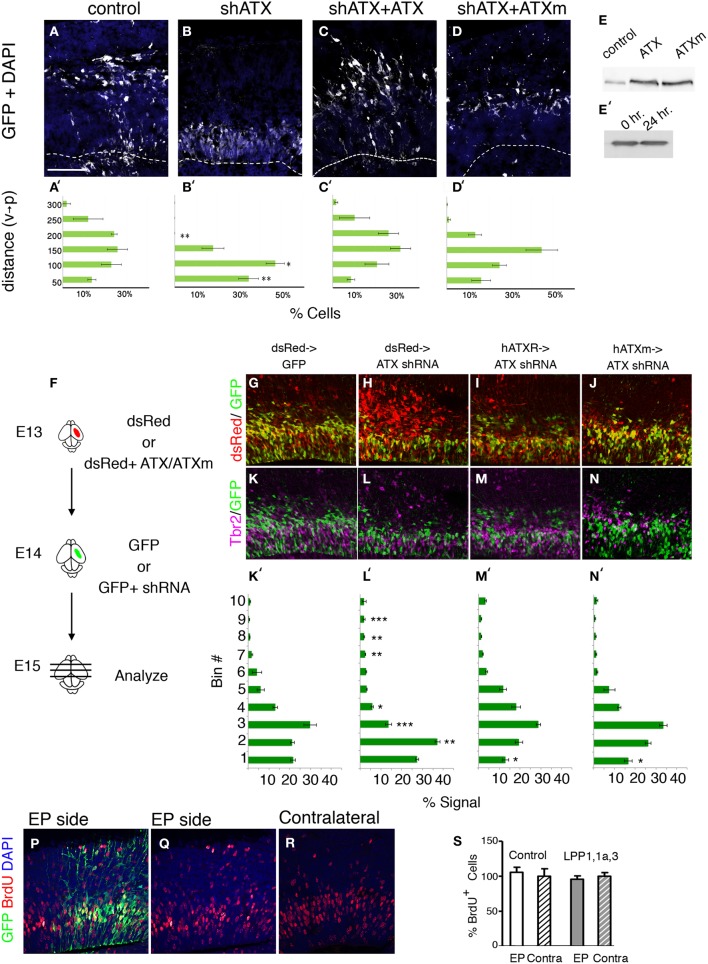
**ATX non-cell autonomous, non-catalytic activity. (A–D′)** E14 mouse embryos were subjected to *ex utero* electroporation with a GFP expression plasmid together with either a control shRNA or *Atx* shRNA. Coronal sections of brains were kept for 2 DIV with condition media. **(B)** Ectopic positioning of cells within the VZ/sVZ can be rescued by addition of external ATX **(C,D)**. **(C–D′)** External addition of ATX **(C,C′)** or mutated ATX (ATXm, **D,D′**) restores normal distribution of knocked-down cells **(B,B′)**. **(E)** ATX constructs were expressed in HEK 293 cells, and media were collected. Albeit ATX is expressed endogenously and secreted (control transfection), transfection of rat ATX or a mutated non-catalytic rat ATX (ATXm) lead to distinctly higher levels of ATX in the media. **(E′)** Western blot of media containing secreted ATX showing that ATX is stable when incubated with brain slices for 24 h. **(F–N) (F)** Experimental design: consecutive electroporation done at E13 and E14. dsRed expressing plasmid was electroporated with or without cDNA encoding human ATX (hATX) or calytically inactive ATX (hATXm) at E13. One day later, GFP alone or GFP and shRNA targeting ATX were injected to the same embryos. **(G–N′)** 24 h after the second injection the brains were collected for analysis. Representative sections from control brains **(G,K)** ATX shRNA treated brains **(H,L)** and brains pretreated with hATX **(I,M)** or mutated hATX **(J,N)** were stained with Tbr2 **(K–N)** and DAPI. **(K′–N′)** Signal recorded along the width of the cortex showing the dispersion of cells along the radial aspect of the cortex in arbitrary bin. Smaller numbered bins are apical. (*n* = 3). **(P–S)** LPP overexpression do not affect progenitors postionining during S phase. LPP1/LPP1a/LPP3 expressing or control vectors where coelectroporated with GFP plasmid *in utero* at E14 and analyzed 24 h later. BrdU labeling was done 1 h prior to the analysis. **(Q–R)** The position of BrdU positive cells in the electroporated (EP side, **Q**) BrdU positive cells on the electroporated side as well as in the non electroporated hemisphere (contralateral, **R**) are shown. **(S)** The relative percentage of BrdU positive, GFP positive cells in the electroporated and contralateral cortical hemispheres is plotted. Size markers: **(A)** 100 μm. Statistical analysis, ^*^p < 0.05, ^**^p < 0.005, ^***^p < 0.001.

During the analysis of brain sections of *Atx* shRNA treated brains, we noted that the position of the knocked down cells differed from the control (Figure 4). This observation was strengthened using both *ex utero* and *in utero* electroporations (Figure 7). We questioned whether the positioning of the cells can be rescued in a non-cell autonomous way. To answer this question *ex vivo*, mouse brain were electroporated *ex utero*, the brain were sectioned, and conditioned media collected from HEK293 cells transfected with either wild type ATX expression construct, or a catalytically inactive mutant, was added to the sectioned brains. The position of *Atx* shRNA treated cells differed from the control in a significant manner (Figures 7B,B′) vs. (Figures 7A,A′), (2/10 bins *p* < 0.01, 1/10 bins *p* < 0.05; *n* ≥ 3 brains for each condition. ANOVA analysis followed by Dunnett's multiple comparison test). Cell positioning was significantly restored using either wild-type ATX (Figure 7C) or mutant ATX (Figure 7D). The mutant protein was somewhat less effective than the wild-type, but the two treatments did not differ in a statistically significant manner. Both proteins were expressed at similar levels (Figure 7E), and the protein was not degraded during the 24 h incubation with the brain slice (Figure 7E′). Next, we questioned whether expression of ATX in earlier born cells can rescue the position of later born cells in which ATX levels were reduced. We have again preformed a consecutive electroporation in which ATX cDNA (as well as mutated ATX or dsRed alone) was introduced to the ventricular zone 1 day prior to the injection of the shRNA. We have presumably allowed the cells to express and secrete the protein prior to the reduction of the mRNA levels in the next wave of proliferating neuroblasts (a scheme is shown in Figure 7F, representative images in Figures 5G–M). We later quantified the location of both populations and found that both ATX expression constructs had similar rescue effects regardless of their catalytic activity (Figures 7M′,N′ in comparison with Figure 7L′). The position of the cells in the ATX shRNA treated brains differed in a statistically significant manner from control in 6 out of 10 arbitrary bins along the width of the cortex (One-Way ANOVA, Dunn's multiple comparison test, *p* < 0.05). The non-cell autonomous rescue experiments did not differ from the control in 9 out of 10 bins, suggesting that the rescue was almost complete. Collectively, these data suggest that ATX regulates cell positioning in the ventricular zone in a non-cell autonomous manner.

Since re-expression of enzymatic deficient ATX was able to rescue cell positioning in VZ, we validated this finding by analyzing the effect of decreased LPA (the synthesis product of enzymatic ATX activity) on VZ neurogenesis. Local LPA concentrations are on the one side controlled via the synthetizing enzyme ATX and on the other side via dephosphorylating enzymes like the LPPs. We therefore electroporated LPP1/LPP1a/LPP3 expressing or control vectors in the VZ of the lateral ventricle wall at E14 and analyzed the pups after 24 h (Figures 7P–S). Neurogenesis was assessed using BrdU 1 h prior to dissection. Quantitative assessment of BrdU -positive cells on the electroporated side as well as in the non electroporated hemisphere revealed no significant difference after electroporation of the control or the LPP1/LPP1a/LPP3 expressing vectors, respectively, corresponding to the catalytic-independent functions of ATX. In addition, there was no obvious difference in the position of transfected cells and/or BrdU labeled cells in the transfected or non-transfected side of the brain.

**Figure 8:**
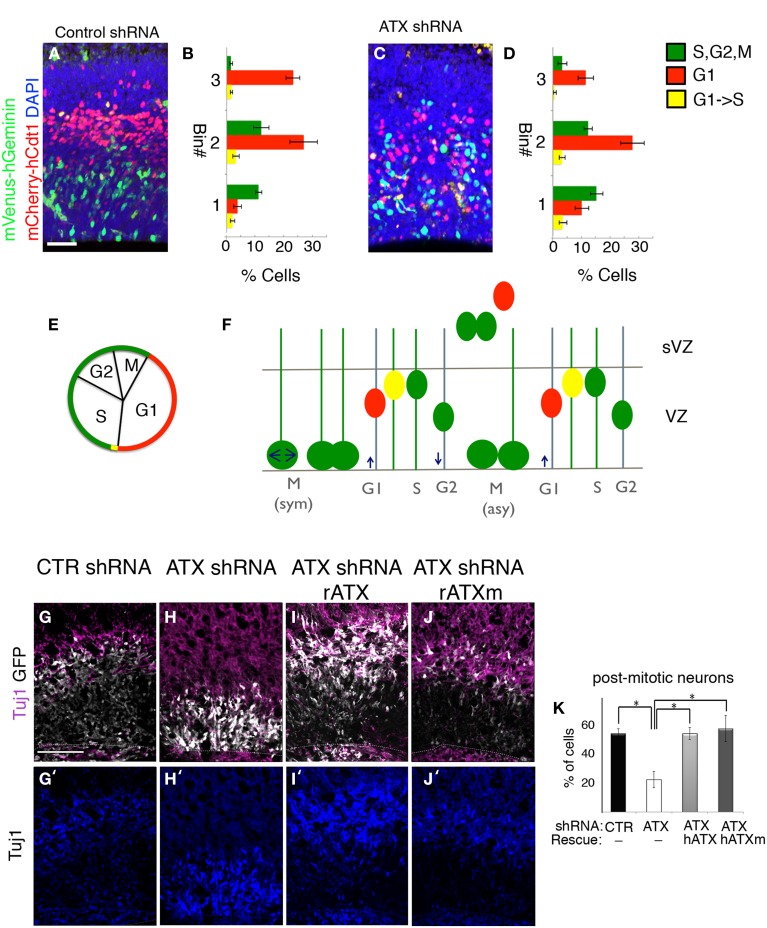
**ATX knock down interferes with progenitors differentiation. (A–D)** Fucci markers (mCherry-hCtd1, mVenus-hGeminin) were electroprated at E13 with control **(A,B)** or ATX shRNA **(C,D)**. Brains were analyzed 24 h later. The location of red cells (G1) Green cells (S,G2,M) and yellow cells (G1->S) was recorded in three arbitrary bins along the width of the cortex. **(E)** Cell cycle correlates with FUCCI markers expression. **(F)** Interkinetic nuclear movement in the ventricular zone (VZ), and cell exiting to the subventricular zone (sVZ) would accumulate the FUCCI markers and alternate colors in correlation with cell cycle. **(G–K)** E14 mouse embryos were subjected to *ex utero* electroporation following organotypic slice culture in condition media, as described earlier. Differentiated post-mitotic neurons with Tuj1 (purple) shows higher colocalization of treated cells (GFP, white) and Tuj1 incontrol cells **(G,G′)** compared with ATX knocked-down cells **(H,H′)**. External addition of both rat catalytic and non-catalytic ATX (rATX, rATXm) restored colocalization of Tuj1 and treated cells **(I–K′)**. **(K)** Statistical analysis, ^*^p < 0.05.

In the ventricular zone, the position of the cell nucleus is tightly linked with cell cycle progression. Furthermore, disruption of the VZ polarity may result in cell cycle defects and interference with neuronal differentiation. Based on our finding of abnormal VZ polarity following knockdown of ATX, we reasoned that ATX might influence cell cycle and proliferation of neuronal progenitors. The effect of *Atx* knockdown on neuronal proliferation in the developing cortex of the mouse, was examined using modified fluorescence ubiquitination cell cycle indicators (FUCCI) (Sakaue-Sawano et al., 2008) (Figures 8A–D). The short-lived fluorescent proteins allow visualizing G1 (red), G1 to S transition (yellow, simultaneous expression of the red and the green fluorescent proteins) and S,G2,M (green) (Figures 8E,F). FUCCI cell cycle reporter plasmids were introduced into E13 developing brains together with either control or *Atx* shRNA. Analysis at E14 revealed that *Atx1* knockdown did not change the percentage of cells in the different stages of the cell cycle in a significant manner (green cells 29 ± 3.1 vs. 34 ± 4.1, red cells 63.1 ± 3.3 vs. 56.7 ± 4.0, and yellow cells 7.8 ± 1.6 vs. 9.2 ± 1.8, in control and *Atx* shRNA treatments respectively, *N* = 8, *Student t-test*). Nevertheless, the position of the different colored cells differed significantly, as can be observed in the representative images (Figures 8A,C). Quantification detected a statistical significant difference in the basal position of cells in G1 (red cells) (*p* < 0.001, *N* = 8, One-Way ANOVA). Polarity at the VZ, which is known to regulate differentiation, was disorganized following ATX depletion. In addition, *Atx* shRNA treated cells were localized within the VZ and displayed a long radial process, a feature of radial glial progenitors. Therefore, we hypothesized that ATX knockdown influences the decision of radial glial to switch from a self-renewing proliferative mode to a differentiation mode. We analyzed the above described using *ex utero* experiments and immunostaining with the postmitotic neuronal marker β-III tubulin (Tuj1). In comparison with control treated cells (Figure 8G), ATX knocked-down cells were more abundant in the VZ and were rarely noted in the IZ (Figure 8H). The percentage of post mitotic Tuj1+GFP+ cells was significantly lower in the *Atx* shRNA treated cells in comparison with control shRNA treated cells (Figures 8G′–J′ quantified in Figure 8K) (*p* < 0.05; *n* ≥ 3 brains for each condition. ANOVA followed by Tukey's HSD test). External addition of either catalytic ATX (Figures 8F,H) or non-catalytic ATX (Figures 8I,J) restored both the localization of the cells as well as the relative percentage of Tuj1+ transfected cells. Compared to the addition of either catalytic or non-catalytic ATX, the percentage of Tuj1+ transfected cells was significantly lower in the ATX knocked-down cells (Figures 8H,K) (*p* < 0.05; *n* ≥ 3 brains for each condition. ANOVA followed by Tukey's HSD test). Collectively, these experiments demonstrated that ATX affects cell positioning and neuronal differentiation in the ventricular zone.

## Discussion

Our unbiased screen for molecules, which participate in non-cell autonomous regulation of neuronal migration revealed *Atx* as a molecule, which is differentially expressed and allowed us to uncover unexpected roles of this molecule in the developing brain. Our results depict a role for ATX in regulation of cell adhesion and cell positioning in neuronal progenitors located in the ventricular zone of the cerebral cortex. We have shown that these activities are (1). cell-autonomous, since the knockdown of *Atx* affects the target cells, but also (2). non-cell autonomous, since knocked down cells could be rescued by addition of external ATX, or by ATX produced by neighboring cells. Remarkably, we observed that the enzymatic activity of ATX was not required to rescue the observed phenotypes. These findings were unexpected since studies in knockout mice revealed that ATX is the major LPA-producing enzyme *in vivo* (Van Meeteren et al., 2006). LPA has been found to affect neural stem cell viability, differentiation and proliferation (Kingsbury et al., 2003; Dottori et al., 2008; Frisca et al., 2013). Nevertheless, previous studies have implicated possible non-catalytic functions for ATX. ATX has been found to induce lung epithelial cell migration *in vitro* through both catalytic-dependent and -independent pathways (Zhao et al., 2011). In addition, ATX promotes changes in cellular adhesion to the extracellular matrix, thereby inducing morphological remodeling in differentiating cultured oligodendrocytes and in CHO-K1 cells which express the P2Y(12) receptor (Fox et al., 2004; Dennis et al., 2008, 2011). Even though our findings emphasize that the role of ATX in neuronal progenitors is predominantly catalytic-independent, a catalytic role of ATX should not be excluded. LPA has essential roles in cortical development, therefore reduced LPA production, due to ATX depletion in neuronal progenitors, might be compensated by other genes involved in LPA homeostasis. Several LPA-regulating genes are expressed in the developing cortex, including phosphatases that degrade LPA such as LPP1 and LPP3 (Giraldi-Guimaraes et al., 2004; Escalante-Alcalde et al., 2007, 2009). Notably, we have shown that increased expression of LPP1, 1a or LPP3 did not affect the proliferation or position of neuroblasts in the ventricular zone. In addition, there are enzymes that produce LPA from different precursors (such as secreted PLA2, Yoshihara et al., 1992; Forlenza et al., 2002; Kurusu et al., 2008). Knockdown of ATX might alter the activity of these genes, thus maintaining normal LPA concentrations. Alternatively, LPA could be supplied from a non-cortical source. In the *ex vivo* experiment, LPA was provided from the cell culture medium. *In vivo*, ATX is highly expressed in the choroid plexus and secreted to the cerebral spinal fluid (CSF) (Sato et al., 2005; Zappaterra et al., 2007). Knockdown of neuronal progenitor-driven ATX could impair both catalytic-dependent and -independent functions. Nevertheless, it should be noted that in our experimental system, CSF-driven ATX did not compensate for the catalytic-independent activities, and therefore effects were observed. However, we cannot exclude the possibility that the LPA derived from the CSF diffuses into the cortex and is sufficient to compensate for the lack of catalytic activity of progenitor-driven ATX.

We uncovered a role for ATX in the regulation of neuronal progenitors. Depletion of ATX disrupted VZ adhesion and polarity establishment. This was documented by the non-polarized expression of several apically-localized proteins and impaired rounded morphology of cells. In addition, we observed proliferation defects and alteration of the cell cycle. Precisely how ATX participates in neuronal progenitor regulation remains to be clarified. We propose that the principal role of ATX is in regulating cellular polarity and attachment to the apical membrane. Changes in proliferation and neurogenesis may stem from altered VZ polarity. Normal adhesion to the apical membrane results in proper cell cycle of progenitors, and the proliferative or neurogenic divisions ensue. Following reduction in ATX levels, adhesion to the apical membrane is diminished. Several lines of evidence established a link between polarity at the VZ, cell cycle progression and cell fate decisions. Adherens junctions act as a self-supporting stem cell niche that maintains cells in a proliferative state (Song et al., 2002; Lien et al., 2006; Stocker and Chenn, 2009; Zhang et al., 2010). Disrupting the maintenance of adherens junctions impairs the Wnt pathway, shortens cell cycle and induces early neuronal differentiation. Both the apically localized Numb and β-catenin are negatively correlated with neuronal differentiation; that is, their constitutive expression results in decreased differentiation, and their reduction leads to decreased cell proliferation (Reiner et al., 2012). Likewise, the PAR complex is positively associated with maintaining a proliferative fate (Cappello et al., 2006; Costa et al., 2008; Bultje et al., 2009). Interkinetic nuclear movement is regulated by the VZ polarity, tightly associated with cell cycle control, and could couple polarity and cell fate decisions (Reiner et al., 2012). We propose that the role of ATX in neuronal progenitors relies on this coordination between polarity at the VZ and cell cycle progress. How is ATX involved in establishment of cellular polarization? Generation of polarity usually requires a signal, which is mediated by a gradient. However, we added external ATX to *Atx* shRNA transfected brains, where the external ATX was distributed equally in the medium. Therefore, ATX might function as a permissive regulator of polarity. Alternatively, ATX might bind to proteins that have polarized distribution and thereby regulate polarity in an instructive manner. Recent studies uncovered that ATX localizes to specific areas in the cell, through binding to either purinergic receptors (Dennis et al., 2011; Zhao et al., 2011) or cell surface integrins (Fulkerson et al., 2011; Hausmann et al., 2011). The interaction with integrins is mediated through the N-terminal somatomedin B-like domain of ATX, while interaction with the P2Y(12) ADP receptor is mediated through the C-terminal part of ATX. These interactions would allow ATX to function in a polarized fashion. In conclusion, this study presents ATX as a crucial regulator of neuronal progenitors. We suggest that ATX regulates polarity, mainly through a catalytic-independent mechanism, and thus influences cell adhesion, positioning and differentiation. ATX was scarcely studied in cortical development, and we hope that future studies will shed light on the underlying mechanisms through which ATX regulates development of the cortex.

## Author contributions

RG, AG, TS, JB, VA, JA, RN, JV and OR were involved in design of the study and writing of the manuscript.

RG, AG, TS, SL, JB, VZ and MS, participated in conducting the experiments, collecting and analysing the data and writing of the experimental results.

### Conflict of interest statement

The authors declare that the research was conducted in the absence of any commercial or financial relationships that could be construed as a potential conflict of interest.
